# Dietary Acrylamide Intake and Risk of Lung Cancer: The Japan Public Health Center Based Prospective Study

**DOI:** 10.3390/nu12082417

**Published:** 2020-08-12

**Authors:** Rong Liu, Ling Zha, Tomotaka Sobue, Tetsuhisa Kitamura, Junko Ishihara, Ayaka Kotemori, Sayaka Ikeda, Norie Sawada, Motoki Iwasaki, Shoichiro Tsugane

**Affiliations:** 1 Division of Environmental Medicine and Population Sciences, Department of Social and Environmental Medicine, Graduate School of Medicine, Osaka University, Suita, Osaka 565-0871, Japan; liur8939@163.com (R.L.); sarin@envi.med.osaka-u.ac.jp (L.Z.); lucky_unatan@yahoo.co.jp (T.K.); sayakaikeda0201@gmail.com (S.I.); 2 Department of Food and Life Science, School of Life and Environmental Science, Azabu University, Sagamihara, Kanagawa 252-5201, Japan; j-ishihara@azabu-u.ac.jp (J.I.); kotemori@azabu-u.ac.jp (A.K.); 3 Epidemiology and Prevention Group, Center for Public Health Sciences, National Cancer Center, Tokyo 104-0045, Japan; nsawada@ncc.go.jp (N.S.); moiwasak@ncc.go.jp (M.I.); stsugane@ncc.go.jp (S.T.)

**Keywords:** dietary acrylamide, lung cancer, cohort, Japan

## Abstract

Acrylamide, which forms in heat-treated foods with high carbohydrate content, is a probable human carcinogen. This study aimed to evaluate the association between dietary acrylamide intake and lung cancer using data from the Japan Public Health Center based Prospective Study. Our study included 85,303 participants who completed a food frequency questionnaire. Cox proportional hazards regression models were used to assess hazard ratios and 95% confidence intervals (CIs) after adjusting for confounders. After 14.3 years and 15.4 years of mean follow-up period, 1187 and 485 lung cancer cases were identified in men and women, respectively. The multivariable-adjusted hazard ratios of 10-µg/day increment in acrylamide intake were 1.01 (95% CI, 0.99–1.02) in men and 0.98 (95% CI, 0.95–1.02) in women. Compared with the lowest quartile of acrylamide intake, the hazard ratios for the highest quartile were 1.13 (95% CI, 0.95–1.33; *p* for trend = 0.12) in men and 1.03 (95% CI, 0.78–1.36; *p* for trend = 0.86) in women in the multivariable-adjusted model. Moreover, there was also no significant association observed in the stratified analysis for histological subtypes of lung cancer. This study demonstrated that dietary acrylamide intake was not associated with increased lung cancer risk in the Japanese population.

## 1. Introduction

In 1994, acrylamide, an important industrial chemical, was classified as probably carcinogenic to humans (Group 2A) by the International Agency for Research on Cancer based on its carcinogenic action in rodents [1]. Until the early 2000s, the primary sources of acrylamide exposure were considered to be specific occupations and smoking [2]. Later, Swedish researchers reported that acrylamide is formed by the Maillard reactions during high-temperature (>120 °C) cooking, mainly in carbohydrate-rich foods, such as French fries and potato crisps, and coffee [3,4,5], reflecting the importance of meals as another source of acrylamide [3]. It is thought that the carcinogenic action of dietary acrylamide occurs in a genotoxic way. Glycidamide, the major metabolite of acrylamide, is able to bind to DNA and cause the formation of DNA adducts [6]. Theoretically, all tissues are targets for carcinogenesis since acrylamide molecules are small and hydrophilic, indicating that it can reach every organ and tissue in the body [7].

Epidemiological studies regarding the relationship between occupational acrylamide exposure and cancer risk have been previously conducted [8,9,10,11,12], and no positive results were observed. In the past years, studies on the association between dietary acrylamide intake and risk of cancers were mainly published in Western countries. According to these studies, positive associations were found only for kidney cancer [13], endometrial and ovarian cancer [14], and postmenopausal estrogen-receptor-positive breast cancer [15].

Potato-based foods, wheat-based products, and coffee are the main food sources of dietary acrylamide in the Western population, while in the Japanese population, the main food sources of dietary acrylamide are coffee and green tea, followed by confectioneries, vegetables, and potatoes [16]. Consequently, the influence of acrylamide intake on cancers in different countries with diverse dietary sources of the chemical needs to be investigated. Recently, several Asian studies evaluated breast, endometrial, ovarian, esophageal, gastric, and colorectal cancer risks in relation to dietary acrylamide intake [16,17,18]. The results showed no positive association, which is consistent with those in Western countries.

To our knowledge, two studies have reported the association between dietary acrylamide exposure and lung cancer [19,20]. However, epidemiological evidence for lung cancer was evaluated inadequately. Therefore, the present study aimed to confirm the potential relationship between dietary acrylamide intake and lung cancer incidence using a Japanese-population-based prospective study.

## 2. Materials and Methods

### 2.1. Study Cohorts and Participants

The Japan Public Health Center based Prospective Study (JPHC) started in 1990 (Cohort I) and 1993 (Cohort II). Its design has been described elsewhere [21]. The JPHC study enrolled 140,420 residents aged 40–69 years in 11 public health center areas across Japan in the baseline survey, using a self-administered lifestyle questionnaire. After 5 years, a follow-up survey including more complete information on food intake frequency was conducted. We applied this 5-year follow-up survey as the starting point of our study.

Participants registered in Tokyo had no available information on cancer incidence, and those registered in Osaka had a different definition of the study population. Therefore, we did not include the participants in these two areas in the current study. Further, we excluded those who did not meet the follow-up criteria and those who failed to complete the 5-year follow-up survey questionnaire. We also excluded participants who died or moved out of the study areas during the time from the baseline survey to the 5-year follow-up survey, those with past history of any cancer, and those who were lost to follow-up. Subsequently, participants providing incomplete information on total energy intake and reporting extremely low or high energy values were also excluded.

All subjects gave their informed consent for inclusion before they participated in the study. The study was conducted in accordance with the Declaration of Helsinki, and the protocol was approved by the Institutional Review Board of the National Cancer Center, Japan (Ethical Approval Code: 2001-021), as well as Osaka University (Ethical Approval Code: 14020-9) and Azabu University (Ethical Approval Code: 2457). 

### 2.2. Assessment of Acrylamide Intake

The estimation of nutrient and food intake was performed using the Food Frequency Questionnaire (FFQ). Information on the common consumption of 147 food items over the past years has been collected [22]. The standard portion sizes were specified in three categories (<0.5 times the standard, same as the standard, and >1.5 times the standard). There were nine frequency categories of food items (never, 1–3 times/month, 1–2 times/week, 3–4 times/week, 5–6 times/week, once/day, 2–3 times/day, 4–6 times/day, and ≥7 times/day). The FFQ was validated by comparison between intake and 28-day weighted dietary records (DRs) as reference in a subcohort of the JPHC Study [22,23,24]. Daily nutrient intake was calculated based on the Fifth Revised and Enlarged Edition of the Standard Tables of Food Composition in Japan (5th FCT) [25].

An acrylamide database [26], developed from measured values of acrylamide content in common Japanese foods [27,28,29,30,31,32,33,34,35,36], was used to estimate acrylamide intake. There were 1878 food items in the 5th FCT. Of these, 282 food items were identified as acrylamide-containing foods. Others were non-acrylamide-containing foods (1276 items) and unclassifiable foods (320 items). Furthermore, since the acrylamide level in the same food is different because of various cooking methods, 39 heat-treated food items were added to the acrylamide-containing foods. Therefore, 321 food items (17% of the total food items) were considered acrylamide-containing foods to estimate the DR-derived acrylamide intake [26]. By linking the measured values of acrylamide content and the list of foods [27,28,29,30,31,32,33,34,35,36], the development of the acrylamide database was completed.

Among the 147 food items of the FFQ, 28 items (19%) were also designated as acrylamide-containing foods. As mentioned previously, the acrylamide level differs between cooking methods. Because the amount of raw food intake in the FFQ is usually used to calculate most nutrient intakes, in calculating the FFQ-derived acrylamide intake, the cooking method for food items listed below needs to be considered, and the proportion of these heat-treated foods calculated from the DR should be used: starchy vegetables (potato, sweet potato), vegetables (onion, bean sprouts, sweet pepper, squash, cabbage, snap beans, broccoli), rice (toast, boiled, or stir-fried), and fried batter. The Spearman’s correlation coefficients for energy-adjusted dietary acrylamide intake between DRs and FFQ were 0.48 and 0.42 in Cohort I and 0.37 and 0.34 in Cohort II in men and women, respectively [26].

### 2.3. Follow-Up and Identification of Cancer Cases

The subjects were followed from the date of the 5-year follow-up survey until 31 December 2013. The annual residential status was confirmed using the residential registry. During the study period, 5022 subjects (5.9%) moved out of the study area, and 13,756 subjects (16.1%) died.

Lung cancer cases were identified from major local hospitals by data linkage with population-based cancer registries. Death certificates were used as a supplementary source of information. All lung cancer cases were defined according to the International Classification of Diseases for Oncology, Third Edition, code C34. At the end of the follow-up period, 1187 lung cancer cases were newly identified in men. Of these, there were 379 adenocarcinomas (M8140, 8211, 8230, 8240, 8246, 8250, 8252, 8254, 8255, 8260, 8310), 323 squamous cell carcinomas (M8070, 8071, 8075, 8560), 140 small-cell lung carcinomas (M8041, 8045), and 345 others and unclassified cases. A total of 485 cases were newly identified in women. Of these, there were 358 adenocarcinomas (M8140, 8211, 8230, 8250, 8252, 8253, 8254, 8255, 8260) and 127 others and unclassified cases.

### 2.4. Statistical Analysis

Analyses in men and women were conducted separately. Accumulated person-years for each subject were estimated from the start of the 5-year follow-up survey to the date of diagnosis of lung cancer, date of death from any cause, date of relocation from the study area, or end of follow-up (31 December 2013), whichever came first. The mean follow-up periods were 14.3 years in men and 15.4 years in women.

The residual method was applied to adjust acrylamide intake using energy intake. Subjects were divided into quartiles (Q1, Q2, Q3, and Q4 groups) based on energy-adjusted acrylamide intake. We used Cox proportional hazards models to estimate the hazard ratios (HRs) and 95% confidence intervals (CIs) of the incidence of lung cancer according to quartiles of energy-adjusted dietary acrylamide intake, with Q1 as the reference group. Trends were measured by assigning ordinal values to quartiles of energy-adjusted acrylamide intake. The characteristics of dietary and nondietary variables between quartiles Q1, Q2, Q3, and Q4 were compared using the Kruskal–Wallis test or chi-squared test as appropriate.

The multivariable model was adjusted for age (5-year age intervals), areas (nine public health center areas), smoking status (never, former, current, or missing), pack-years (<20, 20 to <30, 30 to <40, 40 to <50, ≥50, or missing), body mass index (14 to <19, 19 to <21, 21 to <23, 23 to <25, 25 to <27, 27 to <30, or 30–40 kg/m^2^ or missing), physical activity (continuous), family history of lung cancer (yes or no), alcohol intake (<150 or ≥150 g/week), and energy-adjusted consumption of vegetables and fruits (continuous). In a sensitivity analysis, we excluded 150 men and 45 women with lung cancer diagnosed in the first 3 years of follow-up. 

Furthermore, the level of acrylamide hemoglobin adducts, a biomarker for exposure to acrylamide, is 4 times higher in smokers than in nonsmokers [37]. To elucidate the interaction effect, we performed subgroup analyses for never, former, and current smokers among men. Because 88% of women did not smoke, a subgroup analysis was conducted only for nonsmokers. All *p*-values were two-sided, with significance set at a *p*-value < 0.05. All statistical analyses were performed using Stata version 15.0 (Stata Corp., College Station, TX, USA).

## 3. Results

**Figure 1:**
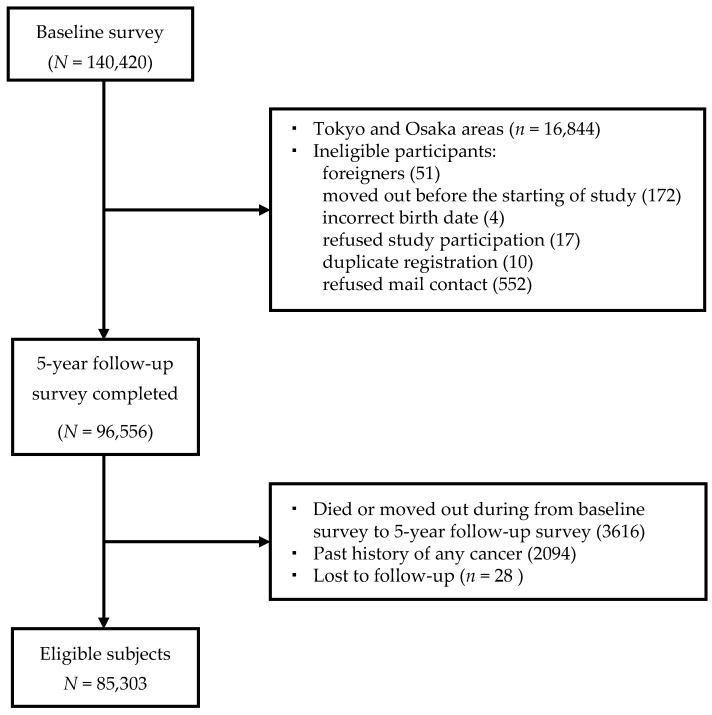
Flowchart of the study.

After the exclusion of ineligible participants, a total of 85,303 subjects (39,982 men and 45,321 women) were included in the current study (Figure 1).

### 3.1. Analysis of Characteristics

Table 1 shows the characteristics of the study population based on acrylamide intake. The mean (standard deviation) daily acrylamide intakes were 6.8 (3.9) µg/day in men and 7.0 (3.7) µg/day in women. The main contributors to total acrylamide intake were coffee (men, 32.0%; women, 23.6%), green tea (men, 20.2%; women, 22.8%), potatoes and starches (men, 10.4%; women, 12.8%), biscuits and cookies (men, 8.0%; women, 12.8%), and vegetables (men, 9.9%; women, 11.5%). Relative to the lowest acrylamide consumption group (Q1), the food and beverage consumption of the highest acrylamide consumption group (Q4) comprised more coffee, green tea, vegetables, potatoes and starches, and biscuits and cookies but less meat, fish, and alcohol. Furthermore, compared with the Q1 group, the Q4 group consisted of younger subjects, a greater proportion of current smokers both in men and women, and a higher pack-years only in men. 

### 3.2. Association between Dietary Acrylamide Intake and Lung Cancer Risk

Table 2 presents the associations between daily acrylamide intake and lung cancer in men. No association was observed between daily acrylamide intake and lung cancer overall (*p* for trend = 0.12). The HR of lung cancer for 10-µg/day increment of acrylamide intake was 1.01 (95% CI, 0.99–1.02). The results of the sensitivity analysis were similar to those of the overall analysis. For never-smokers, there were decreased HRs in the Q2, Q3, and Q4 groups, but the 95% CIs were wide. Meanwhile, no evidence of a decreased risk of lung cancer (HR, 0.98; 95% CI, 0.93–1.02) was observed when acrylamide intake was treated as a continuous variable. For former and current smokers, acrylamide was not significantly related to an increased risk of lung cancer. In addition, no associations were observed in the analysis of any histological subtype of lung cancer.

Table 3 shows the results of daily acrylamide intake and risk of lung cancer in women. Acrylamide intake was not associated with lung cancer risk by increasing acrylamide intake both in the overall analysis (*p* for trend = 0.86) and sensitivity analysis (*p* for trend = 0.86). For never-smokers, there were increased HRs in the Q2 and Q3 groups, but the results were not significant. When acrylamide intake was considered a continuous variable, the HR was 0.98 (95% CI, 0.95–1.01). In addition, a null association was also observed in the analysis of adenocarcinoma of lung cancer. Limited by the small number of former and current smokers and other histological subtypes of lung cancer, further subgroup analysis was not performed.

## 4. Discussion

Based on this large-scale prospective cohort study, we did not find any association between dietary acrylamide intake and overall risk of lung cancer in both men and women. 

One previous study among Finnish male smokers showed a positive association between dietary acrylamide intake and risk of lung cancer with a multivariable relative risk of 1.18 (95% CI, 1.01–1.38) in the highest vs. lowest level [20]. In our study, the HR for high vs. low level of acrylamide intake was 1.13 (95% CI, 0.95–1.33). One reason for our null association could be that Japanese men had much lower acrylamide intake relative to Finnish men. The median acrylamide intakes of the lowest level were 21.9 and 3.0 µg/day for Finish and Japanese male smokers, and the median acrylamide intakes of the highest level were 55.7 and 11.2 µg/day, respectively. 

Another study in the Netherlands reported that acrylamide intake was inversely associated with lung cancer risk in women but was not associated in men. The overall risk of lung cancer has statistically significantly decreased with increased acrylamide intake in women. The HRs were 0.82 (95% CI, 0.69–0.96) for a 10-µg/day increment of acrylamide intake and 0.45 (95% CI, 0.27–0.76; *p* for trend = 0.01) for the highest vs. lowest quintile of acrylamide intake [19]. The authors suggested that acrylamide may affect the hormonal balance, supported by postmenopausal hormone treatment associated with decreased lung cancer risk in some studies. In contrast, the results in all men in the Netherlands were similar to those of our study. For male never-smokers, the risk of lung cancer in the former study was increased in the second and third tertiles compared to the lowest tertile. However, in our study, we observed decreased risk across the intake quartiles, although *p* for trend was not significant.

The average acrylamide intake was 21.7 µg/day in the Netherlands Cohort Study on Diet and Cancer and 26.2 µg/day in the European Prospective Investigation into Cancer and Nutrition [38], with both being much higher than that in our study. Green tea is a specific source of acrylamide exposure in the Japanese population, contributing substantially to the total exposure. One study in Japan divided subjects into green tea drinkers and nondrinkers to investigate the relationship between dietary acrylamide intake and risk of digestive system cancer, but no association was found [18]. To date, three Japanese studies on the relationship between dietary acrylamide intake and cancer risk have been reported [16,17,18]. These studies provided evidence that acrylamide intake was not associated with increased risk of breast, endometrial, ovarian, esophageal, gastric, and colorectal cancer in Japanese individuals. Because of differences in average acrylamide intake and common null association between acrylamide intake and most cancers, there might be no substantial difference between Asian and Western populations in sensitivity to acrylamide intake.

Our study has some strengths. This study has a prospective cohort study design. The avoidance of recall bias in exposure was made by collecting data before the diagnosis of lung cancer. The sample size of this study was large, with participants selected from the general population. The cumulative incidence rates of lung cancer among participants from 1995–2013 were similar to that of the general Japanese population. In addition, the proportion of lung cancer cases ascertained by death certificate only was 2.6%, proving sufficient quality of the cancer registries in this study.

There are some limitations in the current study. First, acrylamide levels differ widely across foods, which may contribute to misclassification of dietary acrylamide intake. We used acrylamide values of foods measured in the 2000s because measured values were not available in the 1990s. However, foods consumed in the 1990s had a higher proportion of beverages but a lower proportion of vegetables than those in the 2000s [34]. Acrylamide values measured in the 2000s may not completely represent foods in the 1990s. Furthermore, FFQ is the only workable way in large-scale epidemiological studies to determine dietary acrylamide intake over a long period, although estimated acrylamide intake depends on the question items and options of the questionnaire. The correlation coefficients of dietary acrylamide intake from the FFQ and 28-day DRs ranged from 0.34 to 0.48 [26]. Second, passive smoking is a risk factor for lung cancer in Japanese women [39]. Since the 5-year follow-up survey did not include related information, we could not further adjust for this risk factor in the model. Finally, unknown confounders may have affected the results, although we had adjusted for possible confounders in the analysis.

## 5. Conclusions

This study did not provide evidence that dietary acrylamide intake has a positive association with lung cancer in the Japanese population, in both men and women.

## Figures and Tables

**Table 1:** Characteristics of participants (*n* = 85,303) in the lung cancer analysis.

Characteristics	Quartiles of Energy-Adjusted Acrylamide Intake								
	Quartile 1		Quartile 2		Quartile 3		Quartile 4		*p*-Value ^1^
**Men**									
Number of participants	9996		9995		9996		9995		
*Dietary variables*									
Acrylamide intake									
Range, µg/day	0.0–4.1		4.1–5.9		5.9–8.6		8.6–54.3		
Mean and SD, µg /day	2.9	(0.8)	5.0	(0.5)	7.1	(0.8)	12.1	(3.7)	
Mean and SD, µg kg body weight^−1^·day^−1^	0.05	(0.04)	0.08	(0.07)	0.11	(0.10)	0.19	(0.24)	
Coffee, g/day	36.6	(52.0)	91.5	(88.0)	167.1	(141.3)	397.8	(350.1)	<0.001
Green tea, g/day	265.1	(301.3)	430.0	(403.2)	524.6	(442.2)	750.9	(706.8)	<0.001
Potatoes and starches, g/day	8.0	(8.0)	13.6	(12.2)	17.2	(15.9)	18.9	(23.6)	<0.001
Biscuits and cookies, g/day	0.5	(0.9)	1.1	(1.6)	1.9	(2.8)	4.1	(7.7)	<0.001
Vegetables, g/day	159.0	(109.8)	191.9	(116.9)	208.1	(128.4)	208.4	(138.4)	<0.001
Fruit, g/day	141.3	(139.6)	174.2	(142.4)	184.8	(147.0)	180.7	(150.3)	<0.001
Meat, g/day	60.9	(47.4)	61.0	(40.0)	61.3	(38.7)	60.7	(38.0)	<0.001
Fish, g/day	87.5	(60.8)	90.2	(52.2)	90.3	(51.8)	84.8	(50.8)	<0.001
Alcohol intake, g/day	280.1	(285.3)	221.1	(240.0)	185.4	(221.1)	134.1	(188.2)	<0.001
Total energy intake, kcal/day	2152.6	(655.4)	2168.1	(640.0)	2187.2	(640.1)	2151.5	(653.7)	<0.001
*Nondietary variables*									
Age at 5-year follow-up study, year	57.4	(7.4)	57.1	(7.8)	56.8	(8.0)	56.0	(8.1)	<0.001
Body mass index, kg/m^2^	23.6	(2.9)	23.7	(2.9)	23.6	(2.8)	23.5	(2.9)	<0.001
Smoking status, %									
Never	35.9		36.4		34.0		28.2		<0.001
Former	18.4		19.1		18.5		16.3		
Current	42.9		42.0		45.1		53.4		
Missing	2.9		2.5		2.5		2.2		
Pack-years, for former and current smokers	35.5	(25.9)	35.3	(19.4)	36.5	(23.5)	39.4	(21.2)	<0.001
Physical activity (METs)	36.8	(10.5)	37.8	(10.2)	37.8	(10.1)	37.5	(10.1)	<0.001
**Women**									
Number of participants	11,331		11,330		11,330		11,330		
*Dietary variables*									
Acrylamide intake									
Range, µg/day	0–4.4		4.4–6.2		6.2–8.7		8.7–69.9		
Mean and SD, µg/day	3.25	(0.9)	5.3	(0.5)	7.35	(0.7)	11.96	(3.3)	
Mean and SD, µg kg body weight^−1^·day^−1^	0.06	(0.1)	0.10	(0.1)	0.14	(0.1)	0.23	(0.2)	
Coffee, g/day	28.7	(43.5)	68.73	(72.3)	118.28	(112.4)	251.39	(260.0)	<0.001
Green tea, g/day	301.88	(325.5)	486.27	(412.7)	589.34	(454.5)	869.41	(759.2)	<0.001
Potatoes and starches, g/day	10.8	(9.2)	17.55	(13.6)	20.73	(17.0)	22.86	(26.0)	<0.001
Biscuits and cookies, g/day	0.95	(1.2)	1.84	(2.0)	3.08	(3.4)	6.33	(8.9)	<0.001
Vegetables, g/day	196.95	(122.9)	224.88	(120.3)	235.16	(128.5)	237.11	(139.3)	<0.001
Fruit, g/day	215.79	(173.6)	237.51	(161.1)	240.17	(159.6)	231.46	(168.1)	<0.001
Meat, g/day	57.06	(43.1)	53.95	(35.1)	52.63	(33.8)	51.67	(33.6)	<0.001
Fish, g/day	85.78	(55.4)	86.49	(46.4)	83.5	(45.6)	78.83	(44.3)	<0.001
Alcohol intake, g/day	16.17	(84.1)	12.43	(57.2)	12.45	(56.7)	12.43	(51.1)	<0.001
Total energy intake, kcal/day	1834.96	(587.2)	1858.17	(551.6)	1883.34	(561.4)	1838.6	(567.8)	<0.001
*Nondietary variables*									
Age at 5-year follow-up study, years	58.67	(7.6)	57.77	(7.9)	56.95	(8.0)	55.58	(8.0)	<0.001
Body mass index, kg/m^2^	23.7	(3.3)	23.6	(3.2)	23.48	(3.2)	23.42	(3.2)	<0.001
Smoking status, %									
Never	87.63		88.72		88.57		85.1		<0.001
Former	0.88		0.94		0.9		1.29		
Current	4.22		4.19		4.58		7.62		
Missing	7.27		6.15		5.95		5.99		
Pack-years, for former and current smokers	22.1	(18.5)	20.2	(14.8)	19.3	(15.1)	21.4	(14.2)	<0.001
Physical activity (METs)	35.94	(9.4)	37.22	(9.0)	37.6	(8.9)	37.68	(9.0)	<0.001

Values are mean ± SD or percentages. ^1^ Kruskal–Wallis test for continuous variables and chi-squared test for categorical variables.

**Table 2:** Hazard ratios (95% confidence intervals) for lung cancer in men according to quartiles of acrylamide intake.

	Quartiles of Energy-Adjusted Acrylamide Intake										
	10 (μg)		Quartile 1 (lowest)		Quartile 2		Quartile 3		Quartile 4 (Highest)		
	HR	(95% CI)	HR	(95% CI)	HR	(95% CI)	HR	(95% CI)	HR	(95% CI)	*p* for Trend
**Overall lung cancer**											
*All men*											
Number of subjects	39,982		9996		9995		9996		9995		
No. of cases/no. of person-years	1187/569,928		278/140,842		264/142,903		287/143,243		358/142,940		
Multivariable model a ^1^	1.01	(0.99–1.02)	1.00	(Reference)	0.96	(0.81–1.14)	1.00	(0.85–1.19)	1.13	(0.95–1.33)	0.12
Multivariable model a (excluding cases <3 years)	1.01	(0.98–1.01)	1.00	(Reference)	0.96	(0.80–1.15)	1.00	(0.83–1.20)	1.12	(0.94–1.34)	0.16
*Never-smokers*											
Number of subjects	13,430		3584		3633		3396		2817		
No. of cases/no. of person-years	156/198,485		44/52,988		38/53,783		42/50,238		32/41,475		
Multivariable model b ^2^	0.98	(0.93–1.02)	1.00	(Reference)	0.78	(0.50–1.21)	0.92	(0.59–1.43)	0.89	(0.56–1.44)	0.82
*Former smoker*											
Number of subjects	7225		1837		1913		1847		1628		
No. of cases/no. of person-years	192/99,827		46/25,158		39/26,520		57/25,543		50/22,605		
Multivariable model c^3^	1.02	(0.99–1.05)	1.00	(Reference)	0.82	(0.54–1.27)	1.23	(0.82–1.83)	1.18	(0.77–1.79)	0.19
*Current smoker*											
Number of subjects	18,321		4283		4198		4507		5333		
No. of cases/no. of person-years	809/258,949		181/59,080		179/59,408		182/64,280		267/76,181		
Multivariable model c	1.01	(0.99–1.03)	1.00	(Reference)	1.03	(0.83–1.23)	0.97	(0.78–1.19)	1.13	(0.92–1.38)	0.30
**Adenocarcinoma**											
No. of cases/no. of person-years	379/569,928		95/140,842		91/142,903		82/143,243		111/142,940		
Multivariable model a	1.01	(0.99–1.03)	1.00	(Reference)	0.94	(0.70–1.26)	0.83	(0.61–1.13)	1.05	(0.79–1.41)	0.87
**Squamous cell lung carcinoma**											
No. of cases/no. of person-years	323/569,928		83/140,842		59/142,903		80/143,243		101/142,940		
Multivariable model a	1.01	(0.99–1.03)	1.00	(Reference)	0.71	(0.50–1.01)	0.91	(0.66–1.25)	0.98	(0.72–1.35)	0.69
**Small-cell lung carcinoma**											
No. of cases/no. of person-years	140/569,928		34/140,842		30/142,903		36/143,243		40/142,940		
Multivariable model a	1.00	(0.96–1.04)	1.00	(Reference)	0.94	(0.57–1.55)	1.11	(0.68–1.79)	1.11	(0.68–1.81)	0.55

Abbreviations: HR = hazard ratio; 95% CI = 95% confidence interval. ^1^ Multivariable model a adjusted for age (5-year age intervals), areas, smoking status (never, former, current, or missing), pack-years (<20, 20 to <30, 30 to <40, 40 to <50, ≥50, or missing), body mass index (14 to <19, 19 to <21, 21 to <23, 23 to <25, 25 to <27, 27 to <30, or 30–40 kg/m^2^ or missing), physical activity (continuous), family history of lung cancer (yes or no), alcohol intake (<150 or ≥150 g/week), and energy-adjusted consumption of vegetables and fruits (continuous). ^2^ Multivariable model b adjusted for age (5-year age intervals), areas, body mass index (14 to <19, 19 to <21, 21 to <23, 23 to <25, 25 to <27, 27 to <30, or 30–40 kg/m^2^ or missing), physical activity (continuous), family history of lung cancer (yes or no), alcohol intake (<150 or ≥150 g/week), and energy-adjusted consumption of vegetables and fruits (continuous). ^3^ Multivariable model c adjusted for age (5-year age intervals), areas, pack-years (<20, 20 to <30, 30 to <40, 40 to <50, ≥50, or missing), body mass index (14 to <19, 19 to <21, 21 to <23, 23 to <25, 25 to <27, 27 to <30, or 30–40 kg/m^2^ or missing), physical activity (continuous), family history of lung cancer (yes or no), alcohol intake (<150 or ≥150 g/week), and energy-adjusted consumption of vegetables and fruits (continuous).

**Table 3:** Hazard ratios (95% confidence intervals) for lung cancer in women according to quartiles of acrylamide intake.

	Quartiles of Energy-Adjusted Acrylamide Intake										
	10 (μg)		Quartile 1 (lowest)		Quartile 2		Quartile 3		Quartile 4 (Highest)		
	HR	(95% CI)	HR	(95% CI)	HR	(95% CI)	HR	(95% CI)	HR	(95% CI)	*p* for Trend
**Overall Lung Cancer**											
*All women*											
Number of subjects	45,321		11,331		11,330		11,330		11,330		
No. of cases/no. of person-years	485/698,086		113/174,917		135/174,528		129/174,527		108/174,114		
Multivariable model a^1^	0.98	(0.95–1.02)	1.00	(Reference)	1.21	(0.94–1.56)	1.19	(0.92–1.54)	1.03	(0.78–1.36)	0.86
Multivariable model a (excluding cases <3 years)	0.97	(0.95–1.01)	1.00	(Reference)	1.20	(0.92–1.56)	1.14	(0.87–1.49)	0.99	(0.74–1.32)	0.86
*Never-smokers*											
Number of subjects	39,658		9929		10,052		10,035		9642		
No. of cases/no. of person-years	406/618,469		94/155,579		118/156,467		117/156,315		77/150,108		
Multivariable model b ^2^	0.98	(0.95–1.01)	1.00	(Reference)	1.28	(0.97–1.68)	1.30	(0.98–1.72)	0.98	(0.71–1.33)	0.98
**Adenocarcinoma**											
No. of cases/no. of person-years	358/698,086		79/174,917		96/174,528		104/174,527		79/174,114		
Multivariable model a	0.97	(0.94–1.01)	1.00	(Reference)	1.21	(0.90–1.64)	1.34	(0.98–1.80)	1.06	(0.77–1.47)	0.57

Abbreviations: HR = hazard ratio; 95% CI = 95% confidence interval. ^1^ Multivariable model a adjusted for age (5-year age intervals), areas, smoking status (never, former, current, or missing), pack-years (<20, 20 to <30, 30 to <40, 40 to <50, ≥50, or missing), body mass index (14 to <19, 19 to <21, 21 to <23, 23 to <25, 25 to <27, 27 to <30, or 30–40 kg/m^2^ or missing), physical activity (continuous), family history of lung cancer (yes or no), alcohol intake (<150 or ≥150 g/week), and energy-adjusted consumption of vegetables and fruits (continuous). ^2^Multivariable model b adjusted for age (5-year age intervals), areas, body mass index (14 to <19, 19 to <21, 21 – <23, 23 to <25, 25 to <27, 27 to <30, or 30–40 kg/m^2^ or missing), physical activity (continuous), family history of lung cancer (yes or no), alcohol intake (<150 or ≥150 g/week), and energy-adjusted consumption of vegetables and fruits (continuous).
